# Recombinant porcine norovirus identified from piglet with diarrhea

**DOI:** 10.1186/1746-6148-8-155

**Published:** 2012-09-03

**Authors:** Quan Shen, Wen Zhang, Shixing Yang, Zhibiao Yang, Yan Chen, Li Cui, Jianguo Zhu, Xiuguo Hua

**Affiliations:** 1 School of Medical Science and Laboratory Medicine, Jiangsu University, 301 Xuefu Road, Zhenjiang, Jiangsu, 212013, People’s Republic of China; 2 Key laboratory of Veterinary Biotechnology, School of Agriculture and Biology, Shanghai JiaoTong University, 800 Dongchuan Road, Shanghai, People’s Republic of China; 3 Food Animal Health Research Program, Ohio Agricultural Research and Development Center, The Ohio State University, Wooster, OH, 44691-40961, USA

**Keywords:** Porcine norovirus, Recombinant, New genotype, Gastroenteritis

## Abstract

**Background:**

Noroviruses (NoVs) are members of the family *Caliciviridae* and are emerging enteric pathogens of humans and animals. Some porcine NoVs are genetically similar to human strains and are classified into GII, like most epidemic human NoVs. So far, PoNoV have been exclusively detected in fecal samples of adult pig without clinical signs.

**Results:**

Result showed that 2 of the 12 evaluated fecal samples were positive for PoNoVs, one of which was positive for PoNoV alone, and the other was coinfected with porcine circovirus and PoNoV. Phylogenetic and recombination analysis showed that the PoNoV positive alone strain was a recombinant new genotype strain. Experimental infection of miniature pigs with fecal suspensions confirmed that this strain can cause gastroenteritis in piglets.

**Conclusion:**

This is the first report that recombinant new genotype PoNoV exised in pig herd of China, which cause diarrhea in pigs in nature condition. This find raised questions about the putative epidemiologic role of PoNoV.

## Background

Noroviruses (NoVs) are members of the family *Caliciviridae* and are emerging enteric pathogens of humans and animals [1,2]. They are small, non-enveloped viruses of 27–38 nm in diameter and possess a single-stranded, positive-sense genomic RNA. NoVs genome is 7.3-7.7 kb in length and contains 3 open reading frames (ORFs) [3]. ORF1 encodes a polyprotein that is autocatalytically cleaved to produce several proteins, including RNA-dependent RNA-polymerase (RdRp) and other non-structural proteins [4]. ORF2 encodes the major capsid protein, and ORF3 encodes for a minor structural protein [5]. NoVs have been isolated from humans and several species of animals, including swine, canine, bovine, murine and lion [6-8]. Based on sequence analyses comprising conserved regions within the RdRp and the capsid genes, NoVs are divided into 5 genogroups (GI-GV) and further subdivided into 27 genotypes [9]. Human NoVs are located in GI, GII and GIV. GII contains 19 genotypes, and porcine strains are found in GII-11, -18 and -19 [10]. Some porcine NoVs are genetically related to human strains and are classified into GII, which contains most epidemic strains of NoVs in human. Some potential NoVs recombinant strains have been identified [10,11], which raises public health concerns regarding their potential for zoonotic transmission. To date, PoNoV have been exclusively detected in fecal samples of adult pig without clinical signs.

## Methods

### Specimens

Twelve fecal samples from piglets with diarrhea and no accurate of etiology were collected from three commercial pig farms in a Shanghai suburb, from May to August, 2009. In order to avoid sample contamination, specimens were obtained directly from the pig anus and disposable materials were used during sampling. Stool samples were freshly collected and immediately converted to 10% (w/v) suspensions in PBS (0.01 M phosphate, pH 7.2-7.4, 0.15 M NaCl,) for RNA and DNA extraction.

### RNA and DNA extraction

RNA was extracted from 200ul of 10% fecal suspension by using the TRIzol reagent (Invitrogen, USA) following the manufacturer’s instructions. RNA pellets were dissolved in 25ul RNase-free water and reverse transcription was performed immediately. DNA was extracted using The QIAamp DNA Stool Mini Kit (QIAGEN, Germany) according to the manufacturer’s instructions.

### RT-PCR or PCR

RT-PCR or PCR assays with different primer sets for the detection of PoNoV, and common viruses that can cause pig diarrhea including porcine circovirus type 2, porcine rotavirus, porcine transmissible gastroenteritis virus, porcine sapovirus, and porcine epidemic diarrhea virus were performed as previously described [12-14]. The amplicons were analyzed by 1% agarose gel electrophoresis in TAE buffer, followed by staining with ethidium bromide (0.5 μg/ml) and visualization under UV light.

### Whole genome amplification

The 3-kb 3^′^end fragment of pNoVs-Ch6 was amplified with primers p290 and VN3T20 [10]. To amplify the remaining sequence, 6 sets of primers (Table 1) were designed based on the entire PoNoV genome sequence (AB126320) available in GenBank. Reverse transcription was carried out at 42°C for 1 h with 1 μl (200 units) of AMV Reverse Transcriptase (TakaRa, Japan) and 1 μl (25 mM) of antisense primer. The PCR parameters of all amplification reactions included an initial incubation at 95°C for 5 min, followed by 39 cycles of denaturation at 94°C for 1 min, annealing for 1 min at a temperature varied according to the Tm of different primers, and extension at 72°C for 1.5 min, with a final incubation at 72°C for 7 min. PCR products were purified using the Axyprep DNA Gel Extraction Kit (AXYGEN, USA). The purified PCR products were ligated into PMD18-T vector (TakaRa, Japan) using T4 DNA ligase (TakaRa, Japan) at 16°C for 2 h. The recombinant plasmid was transformed into DH5α competent Escherichia coli cells (TakaRa, Japan). Three of the positive clones were sequenced.

**Table 1:** Primers designed to amplify the remaining sequence of pNoVs-Ch6

**Primer name**	**Nucleotide position**	**Nucleotide sequence (5**^**′**^**-3**^**′**^**)**
PNoV1F	725-744	ACCTGTCCCTACAGTGGATG
PNoV1R	1494-1513	ATATGAGTTTTGCCTATACC
PNoV2F	1404-1425	ATCTCAGCAGCCAGGTCGCTCC
PNoV2R	2154-2173	AACCCATACCTATTGTCAGC
PNoV3F	2107-2126	TCTACAACTTCGATGTGGAC
PNoV3R	2826-2845	TGACGGAGTCTCATCTCATC
PNoV4F	2744-2762	CGAGGAGTACCTCCGTGAC
PNoV4R	3552-3571	TGATTCTCACCACCCTCCAG
PNoV5F	3525-3543	ACTCAGGGTCCTGATGGTG
PNoV5R	4298-4318	TGCCTCTCTGTTGGGTGGAG
PNoV6F	4261-4279	ATACCTACCACTTTGATGC
PNoV6R	4884-4902	ATGAGGCTTCTCCCAGCAGG
5^′^endF	1-23	GTGAAATGAAGATGGCGTCTAAC
5^′^endR	761-780	ATAGAATCACGTTGGCAACC
3^′^endF1(external forward primer)	7009-7031	ACTGGAATGGCACGAGATACTGG
5^′^endF2(internal forward primer)	7193-7212	ACTCTATGGGTACCTCTAG
5^′^endR		TTTTTTTTT

Position and nucleotide sequence of oligonucleotide primers for PCR. The nucleotide position is in accordance with AB126320. In the primer names, F and R mean sense primer and anti-sense primer, respectively.

### Sequence and recombination analysis

**Figure 1:**
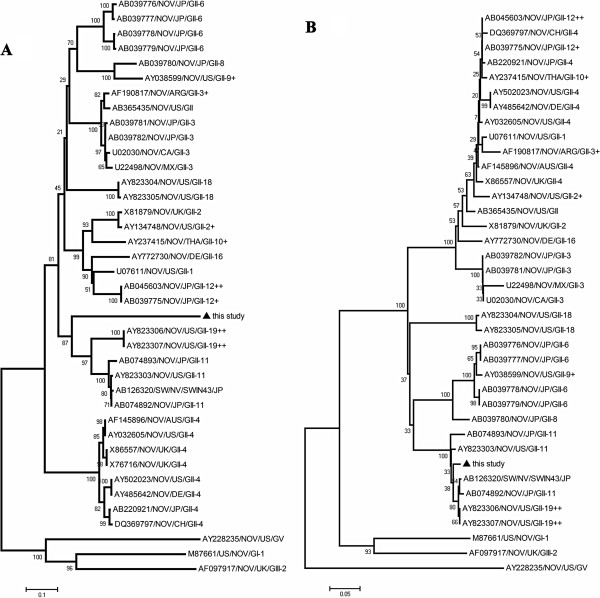
**Phylogenetic analysis of the amino acid sequences of pNoVs-Ch6 and other NoVs. **Bootstrap values, expressed as percentages of 1000 replications, are given at the branch points. GenBank accession numbers for the reference strains are marked at each branch points. Tree (**A**) was constructed based on the complete capsid region. Tree (**B**) was constructed based on the RdRp region. Strains designation follows the outlines of Wang et al. [10] and Zheng et al. [9].

Similarity searches of the sequences were carried out in BLAST (http://www.ncbi.nlm.nih.gov/BLAST/). After a multiple alignment with CLUSTAL W (version 1.4), the phylogenetic relationship of the strains in the present study and the reference isolates were assessed using the software MEGA Version 4.0. For analysis in MEGA, Jukescantor (JC) distance was utilized, employing the Neighbor joining (NJ) algorithm [15]. The reliability of different phylogenetic groupings was evaluated by using the bootstrap test (1000 bootstrap replications) available in MEGA. The identification of recombinants was performed by using the Recombination Detection Program (http://darwin.uvigo.es/rdp/rdp.html) [16]. Prototype NoVs strains used as references in the analysis and their GenBank accession numbers and source of origin are marked in the phylogenetic tree (Figure 1).

### Animals

Eight 15-day old specific-pathogen-free (SPF) miniature pigs were purchased from Experimental Animal Center, School of Agricultural and Biology, Shanghai Jiaotong University (Shanghai, China), and maintained in a pathogen-free animal facility. This animal experiment was approved by the IACUC with a permit number of IACUC-2009-S-0202, which complies with the National Institute of Health Guide for the Care and Use of Laboratory Animals.

### Experimental infection

The pNoVs-Ch6 fecal specimen was converted to 20% (wt/vol) suspensions in phosphate-buffered saline (PBS) (0.01 M, pH 7.4) and clarified by centrifugation at 10,000 g for 10 min. The supernatant was then subjected to purification by passage through microfilters with a pore size of 0.22 μm (Millipore, Japan) to remove possible bacteria and parasites. A 1.5 ml aliquot of supernatant was used to infect five 15-day old piglets through oral inoculation. Another three piglets were only inoculated with PBS as controls. The piglets were investigated every 4 hours during the first day, and fecal samples were collected from each piglet every day. For the experimental group, one piglet was euthanized at post-inoculation day (PID) 1, 2, 4, 6, 10; meanwhile, one was euthanized at PID 1, 4, 10 for the control group. The intestinal contents were collected for RNA testing using RT-PCR, and intestinal segments were excised and immediately immersed in the different fixatives for histological examination. Tissues for histological examination were fixed in 10% neutral buffered formalin, routinely processed, sectioned at a thickness of 7 μm, and stained with hematoxylin and eosin. The tissue sections were examined and compared with negative controls.

## Results

### Result of PoNoV detection

Result of detection indicated that two of the fecal samples were positive for PoNoV, one of which was coinfected with porcine circovirus. The others were negative for PoNoV, porcine circovirus type 2, porcine rotavirus, porcine transmissible gastroenteritis virus, porcine sapovirus, and porcine epidemic diarrhea virus. The fecal sample that was positive for only PoNoV was selected for further genome amplification and experimental infection.

### Genome organization and recombination analysis

The entire genome of this PoNoV strain (named pNoVs-Ch6, GenBank accession number: HQ392821) consisted of 7531 nucleotides, excluding the poly (A) tail. Similar to previously reported NoVs strains, pNoVs-Ch6 has three open reading frames (ORFs), encoding 1693, 547 and 253 amino acids (aa), respectively. It shared 88% nucleotide homology with AB126320 over the entire genome sequence. The highest nucleotide homology over the RdRp sequence was shared with AB126320 (89%) and then with an American porcine strain AY823303 (88%).

**Figure 2:**
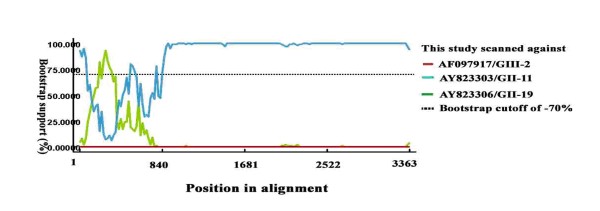
**Identification of recombinant pNoVs-Ch6 strain. **BOOTSCAN evidence for the recombination origin on the basis of pairwise distance, modeled with a window size 200, step size 25, and 100 Bootstrap replicates.

A phylogenetic tree based on the predicted amino acid sequence of the complete capsid region showed that pNoVs-Ch6 was separated from known porcine GII strains (GII-11, GII-18 and GII-19) forming a new branch by itself (Figure 1A), which suggested that this strain may represent a novel genotype in GII group. However, phylogenetic analysis based on the RdRp region gave a different grouping result, where pNoVs-Ch6 was grouped into the GII-11 cluster (Figure 1B). This finding suggested that this virus strain may simultaneously be a recombinant. To confirm the finding and detect the breakpoint where the recombination event occurred, we performed recombination analysis based on 3′end RdRp and the capsid sequence of pNoVs-Ch6 as the query sequence, and AY823303/GII-11 and AY823306/GII-19 as the background sequences using RPD software. Results indicated that the breakpoint located in the RdRp-capsid junction region, and the major parent was AY823303 (Figure 2). The minor parent was not found which may be due to the limited number of NoVs sequences available in GenBank, though AY823306 had a high similarity with the query strain over a short fragment (Figure 2).

### Experimental infection of miniature pigs with PoNoV positive fecal suspensions

**Figure 3:**
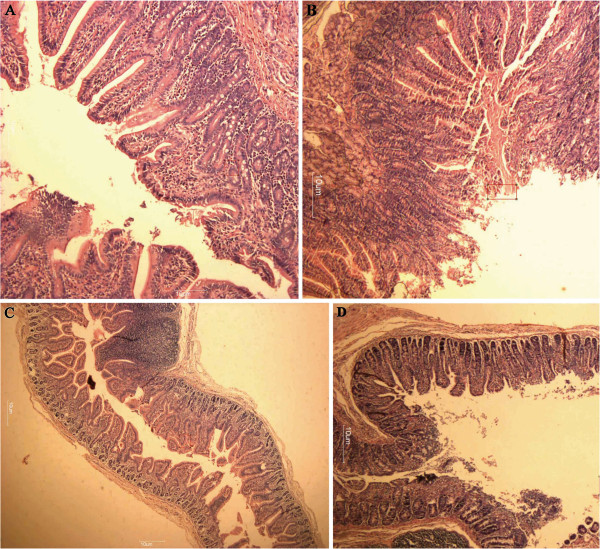
**Histological lesions in the duodenum or jejunum of piglets following oral inoculation with NoVs or PBS. **Hematoxylin and eosin stain. **A**, Normal-appearing, long villi of the duodenum from a PBS-inoculated pig; **B**, Villi of the duodenum showing mild villous atrophy from the NoVs-inoculated pig; **C**, Normal-appearing, long villi of the jejunum from a PBS-inoculated pig; **D**, Villi of the jejunum showing mild villous atrophy from the NoVs inoculated pig. Bar = 10 μm.

Our results showed that all of the five piglets in the experimental group indicated evident symptoms of mild to moderate diarrhea at 0.5-1 PID. The clinical signs persisted for 2 to 6 days, and no piglet died. All of the fecal samples and intestinal contents of the experimental group piglets were positive for NoVs RNA. Mild to moderate villous atrophy, mild to moderate and multifocal villous fusion were observed in the small intestine of experimental group pigs that were euthanatized from PID 6 to PID 10 (Figure 3). The three piglets in the control groups showed no clinical symptoms and tested negative for NoVs RNA.

## Discussion

NoVs has been detected in a wide range of species, including humans, porcine, mice, cows and lions. Because PoNoV are genetically and antigenically related to human GII NoVs, there are public health concerns of potential cross-species transmission and animal reservoirs for NoVs related to human NoVs [10,17]. So far, PoNoV have been exclusively detected in fecal samples of adult pig without clinical signs [18,19], though in experiments with gnotobiotic piglets mild diarrhea occurred [10]. In the current study, gastroenteritis in piglets caused by a PoNoV strain was reported. Experimental infection of miniature pigs with PoNoV positive fecal suspensions confirmed that this virus strain can cause gastroenteritis in pigs. To our knowledge, this study is the first identification of a PoNoV strain that cause piglets gastroenteritis under near natural conditions.

The genome of RNA viruses often undergoes recombination and segmentation [20].

Recombination is a driving force of viral evolution and has been reported for many single-stranded RNA viruses, including NoVs. It has been reported that the recombination of influenza virus leads to generation of a new strain and increases the biological fitness of the virus and its pathogenicity [1].

Previous reports have shown that, in the genome of NoVs, recombination mostly occurs at the junction point of ORF1 and ORF2 which is referred to as ‘hot spot’ [21-23]. Results of phylogenetic analysis and recombination identification showed that pNoVs-Ch6 was a recombinant strain, and the breakpoint located in the RdRp-capsid junction region like most other NoVs recombination events. To the best of our knowledge, this is the first report of recombinant PoNoV in China.

The detection of PoNoV that can cause gastroenteritis in piglets from commercial pig farms under natural conditions, on one hand, raises questions about the putative epidemiologic role of PoNoV, and, on the other hand, this kind of infectious strain also can be used to improve research such as virus replication, vaccine development, which has been impeded by the lack of a cell culture system for the propagation of human NoVs. Whether the recombination increased the virulence of PoNoV still needs further research.

## Conclusion

In conclusion, the results in this study demonstrated that recombinant new genotype PoNoV exised in pig herd of China, which cause diarrhea in pigs in nature condition. This find raised questions about the putative epidemiologic role of PoNoV.

## Authors' contributions

Author Quan Shen, Wen Zhang, Shixing Yang, Yan Chen, Zhibiao Yang and Li Cui performed the experiments. Author Xiuguo Hua, Quan Shen and Wen Zhang designed the experiments. Quan Shen, and Wen Zhang performed the analysis and interpretation of data. Quan Shen wrote the manuscript. All the author approve submission of this manuscript to BMC Veterinary Research.
